# CGI: Java Software for Mapping and Visualizing Data from Array-based Comparative Genomic Hybridization and Expression Profiling

**Published:** 2007-10-06

**Authors:** Joyce Xiuweu-Xu Gu, Michael Yang Wei, Pulivarthi H. Rao, Ching C. Lau, Sanjiv Behl, Tsz-Kwong Man

**Affiliations:** 1 Department of Pediatrics, Baylor College of Medicine and Texas Children’s Cancer Center, Houston, Texas; 2 Department of Computer Science, University of Houston, Victoria, Texas

**Keywords:** aCGH, expression profiling, visualization, correlation, and data integration

## Abstract

With the increasing application of various genomic technologies in biomedical research, there is a need to integrate these data to correlate candidate genes/regions that are identified by different genomic platforms. Although there are tools that can analyze data from individual platforms, essential software for integration of genomic data is still lacking. Here, we present a novel Java-based program called CGI (Cytogenetics-Genomics Integrator) that matches the BAC clones from array-based comparative genomic hybridization (aCGH) to genes from RNA expression profiling datasets. The matching is computed via a fast, backend MySQL database containing UCSC Genome Browser annotations. This program also provides an easy-to-use graphical user interface for visualizing and summarizing the correlation of DNA copy number changes and RNA expression patterns from a set of experiments. In addition, CGI uses a Java applet to display the copy number values of a specific BAC clone in aCGH experiments side by side with the expression levels of genes that are mapped back to that BAC clone from the microarray experiments. The CGI program is built on top of extensible, reusable graphic components specifically designed for biologists. It is cross-platform compatible and the source code is freely available under the General Public License.

## Introduction

With the advent of genomic technologies, DNA and RNA-based microarrays are becoming more accessible to biomedical researchers. One of the common DNA platforms is array-based Comparative Genomic Hybridization (aCGH), which can identify DNA copy number aberrations in the genome (Pinkel, 1998; Man, 2004). There are many software tools that have been developed to analyze aCGH data (Jong 2004; Margolin, 2005; Chen, 2005; Cheung, 2005; Price, 2005; Kim, 2005) and expression microarray data (Sykacek, 2005; Shamir, 2005; Saraiya, 2005; Li, 2001; Vaquerizas, 2005; Bumm, 2002; Saeed, 2003); however, no tool is currently available for the biologist to integrate these two types of data. One of the main challenges is that once the significant BAC clones or genes are identified, it is very difficult to correlate the DNA copy number and RNA expression results. This is because the significant genes may not lie within the corresponding BAC clones even though they are located in the same chromosomal region. Therefore, a more precise method of matching is needed in order to properly correlate these two types of data.

A typical way to perform the matching is to manually search the UCSC Genome Browser (http://genome.ucsc.edu/) to make sure the significant genes lie within the significant BAC clones. However, this type of manual search is very laborious and error prone if the numbers of BAC clones and genes are large. Thus, it is important to develop a user friendly and flexible tool that can match, correlate and display the aCGH and expression profiling data. Since it is common to identify hundreds to thousands of significant genes by either expression profiling or aCGH experiments, our program can further assist researchers to select genes that are found to be significant by both types of experiments, or genes that may not be identified by using either type of technique alone.

To address this issue, we developed a Java-based, stand-alone program that uses MySQL database (http://www.mysql.com) as a backend to store the BAC clones and gene information downloaded from UCSC database. This information is used to match the user-provided BAC clones in aCGH experiments and genes in expression profiling experiments. After that, the correlation coefficients and p-values of the matched BAC clone-gene pairs will then be computed and displayed in various formats for data visualization and comparison.

## Software Designs

The CGI software is based on an object-oriented framework designed to conduct searches for features/genes in RNA expression-profiling experiments that mapped back to corresponding BAC clones in aCGH experiments. The program combines bioinformatic data matching from databases with simple correlation analysis. The software is organized into three functional modules (Data, Annotation, and Correlation). The Data module contains DNA copy number and RNA expression data and links them with the Annotation module by interacting with the MySQL database that holds a variety of different types of genomic information including chromosomal localization, Unigene ID, and gene annotation data. Information in the database is used to match the BAC clones and the genes provided by the users. The Correlation module calculates the Pearson correlation coefficients and p-values between the DNA copy numbers and expression values of matched BAC clone-gene pairs in different experiments. It also displays DNA copy numbers of a specific BAC clone in different aCGH experiments and the associated gene expression values in microarray experiments for easy data visualization and comparison.

## Data Importing

**Figure 1:**
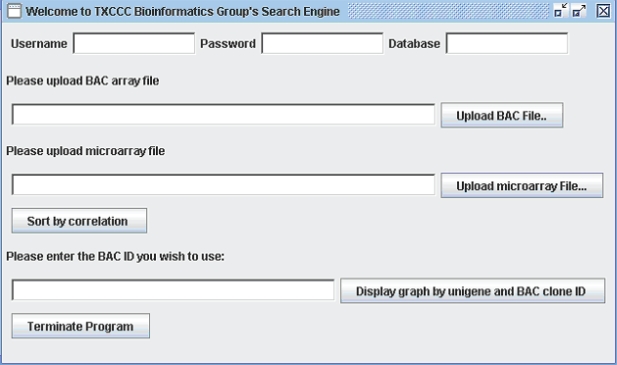
Graphic user interface of CGI for data importing and analysis.

A simple graphical user interface (GUI) prompts users to enter user name, password, database name, and the locations of the aCGH and RNA expression-profiling files (Fig. 1). The aCGH file contains FISH-mapped BAC Clone IDs, cytobands, and normalized log ratios representing DNA copy numbers from aCGH experiments. The RNA expression-profiling file contains Unigene IDs, gene symbols, and log-ratios (dual channel arrays) or log intensities (Affymetrix or oligo-based arrays) of gene expressions in a set of experiments involving identical cases as in the aCGH experiments.

## Data Querying and Mining

The program offers two ways to query the data. First, BAC Clone IDs in an aCGH input file are used to query the MySQL database, which stores data downloaded from the UCSC database at URL:http://genome.ucsc.edu/cgi-bin/hgTables-fishClone and uniGene_2 tables. The two tables are first downloaded by the user and imported to the MySQL databases as described in the installation manual (see supplemental information). The Unigene IDs of the genes that reside in each BAC clone in the aCGH input file are retrieved based on chromosome number and their physical locations by SQL commands. Secondly, these Unigene IDs are used to match with the features/genes provided in a RNA expression-profiling input file, so that the matched BAC clone-gene pairs will be identified. The DNA copy numbers and gene expression values of the matched BAC clone-gene pairs will then be extracted from the input files and their Pearson correlation coefficients and p-values are computed by an internal correlation functions. Finally, the correlation coefficients and p-values of the BAC clone-gene pairs will be tabulated together with their BAC Clone IDs, cytobands, Unigene IDs, and gene symbols provided by the input files. If there are multiple genes within a BAC clone, the program will replicate the DNA copy number data of that BAC clone and correlate with the expression data of each of the other genes that are mapped to the BAC clone.

## Data Visualization

**Figure 2:**
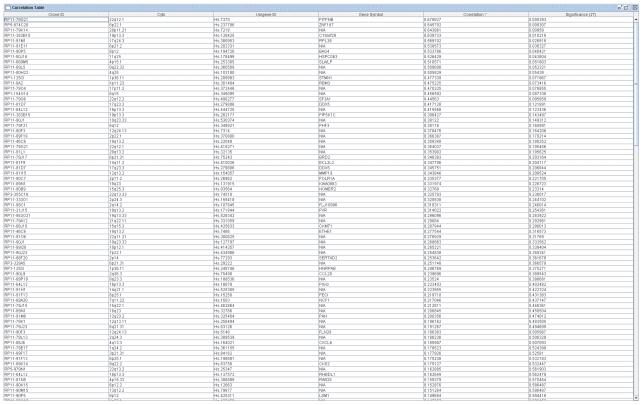
Table view to display the correlations of the matched BAC clones in aCGH and genes in expression microarray experiments.

**Figure 3:**
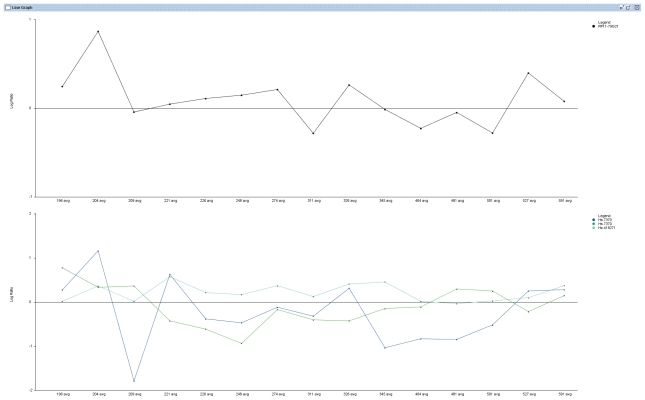
Line graph viewer. The top panel is the copy number changes of a BAC clone in a set of aCGH experiments. The bottom panel is the gene expression values of three corresponding genes that matched the BAC clone in expression microarray experiments using the same experimental cases.

**Figure 4:**
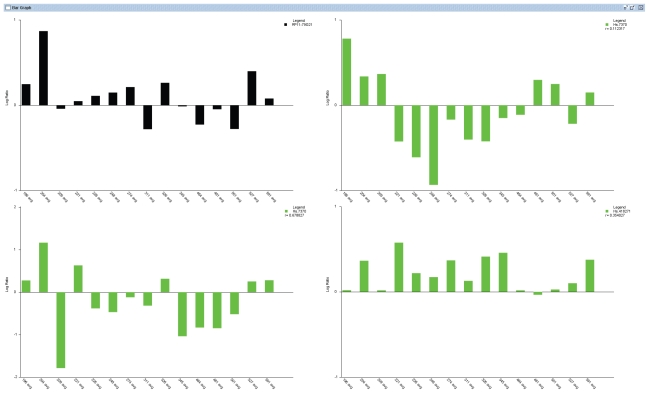
Bar graphic viewer. The black bar graph shows DNA copy numbers (on the Y-axis) of the BAC clone from 15 different experiments (numbered on X-axis). The relative gene expression values (on the Y-axis) of three corresponding genes mapped to the BAC in expression array experiments were displayed in three separate green bar graphs for clearer visualization.

CGI uses a correlation table to display a global overview of BAC Clone ID, Cytoband, their corresponding Unigene IDs, gene symbols and Pearson correlation coefficients and p-values of the matched BAC clones and features/genes. The table view is very flexible and the data in the table can be sorted dynamically in an ascending or descending order based on the correlation coefficients, BAC Clone ID, cytoband location, Unigene ID, etc (Fig. 2). It can also change the order of the columns to display different views according to user’s preference. Besides the table view, users can also visualize in detail the DNA copy number of a specific BAC Clone and the expression of its associated genes by entering the BAC Clone ID into the text box provided in the GUI (Fig. 1). The CGI program will display two graphic windows if the input BAC Clone ID matches one or more Clone IDs in the correlation table. One window displays three line graphs representing the DNA copy number changes of the queried BAC Clone in aCGH experiments and the expression values of its associated genes in RNA expression-profiling experiments (Fig. 3). The second window displays the DNA copy number data and RNA expression data as separate bar graphs for better visualization of the individual experiments if the number of matched genes is high (Fig. 4). This function provides a graphical visualization of the correlation between a BAC clone and its matching genes/features.

## Application

To test this program, we have analyzed a previously published dataset that contains data from both aCGH arrays (Man, 2004) and cDNA microarrays (Man, 2005) of a set of pediatric osteosarcoma patients. We found several genes with RNA expressions correlating with the DNA copy numbers in the corresponding BAC clones (r >0.5, n = 15, p < 0.05, Fig. 2). One of the highly correlated genes (ZNF187) is mapped back to the BAC clone RP5-874C20, which is one of the most frequently amplified regions (6p21.1) in osteosarcoma (Man, 2004). ZNF187 or SRE-ZBP is induced by serum response and may regulate oncogene c-fos by binding to its serum response element (Attar, 1992). We have validated matching and correlation results of CGI by manually searching the UCSC genome browser to confirm the match between BAC Clone ID and Unigene ID, and recalculated their correlation coefficients using an independent method.

## Discussion

We have developed the CGI program, which provides a simple yet powerful tool for matching, correlating, and visualizing aCGH and gene expression-profiling results simultaneously in multiple experiments. This tool is useful because it correlates the results from DNA profiling with those from RNA expression-profiling experiments in order to identify genes that are important at both DNA and RNA levels. The genes that are significantly altered in both sets of experiments add more confidence to the biological significance of these genes and therefore warrant further investigation. It also alleviates the need for manual matching between BAC clones on the aCGH arrays and the features in gene expression arrays using public databases. For data analysis, it provides a visualization tool and correlation calculations with an interactive and flexible interface. We have also implemented error detection routines to handle the database connection, e.g. user needs to enter username, password, and database name for secure connection. The number of experiments in the input files are also checked to ensure comparability of the data. This software was implemented in an object-oriented language, Java, to ensure portability across different operating systems. It is a stand-alone program, which is designed for users to install and run on their own local machine. Therefore, unlike other web-based analytical tools, the users do not need any server support, and are not affected by Internet traffic, server-side problems and downtime. Instead of using flat file data storage, the CGI program also provides fast data access and transfer from MySQL database, which is freely available via the web site (http://www.MySQL.com).

Different from some analytical tools, such as BioConductor (www.bioconductor.org), which uses command line interface, the CGI program uses an easy-to-use and intuitive graphical interface for bench biologists to perform the analysis without any prior computational background. A detailed description on how to install the databases and program is also provided in the supplementary information. The software framework that we employ supports the development of more sophisticated visualization and analytical functions in the future through its open API for Java-based plug-ins. The program is coded in Java reusable object classes, thus promoting a rapid development of future program extensions.

Two similar efforts of comparing aCGH and expression-profiling have been published recently. Kingsley et al have recently developed a web-based system, Magellan, which explores the quantitative relationship between aCGH and mRNA expression data (Kingsley, 2006). Magellan computes the relationship of aCGH and expression based on common annotation values between the two sets of experiments. Shankar et al has also developed a program mainly to visualize aCGH and expression data (Shankar, 2006). In contrast to these other two programs, the CGI program is standalone program, which does not require Internet connection and is not affected by the server-side problem. In addition to visualizing the data, the main strength of CGI is to provide an easy-to-use interface for fast matching and correlation of these two types of genomic data using a relational database. Once these candidate genes are identified, they can be subjected to additional analyses using other existing analytical tools. Since the software is developed in the object-oriented language Java, it can interact with other programs currently available for aCGH and microarray analysis, such as the BioConductor packages. It is straightforward to include other computational algorithms to extend the analytical capability of the program. The modular design of this program also adds flexibility and extensibility for the development of more functions and plug-ins in the future. In summary, we have developed an easy-to-use program CGI to map, correlate, and visualize aCGH and expression profiling data.
